# Associations Between Early-Life Exposure to Different Antibiotic Agents and the Risks of Autism Spectrum Disorder and Attention-Deficit/Hyperactivity Disorder: A Nationwide Population-Based Study

**DOI:** 10.3390/pharmaceutics18091088

**Published:** 2026-08-28

**Authors:** Hao-Yuan Lee, Yu-Chia Chang, Chyi-Liang Chen, Shu-Hua Ko, Yu-Ling Huang, Shang-Po Shen, Yu-Lung Hsu, Wen-Yuan Lee, Hung-Chih Lin

**Affiliations:** 1 Department of Nursing, Jen-Teh Junior College of Medicine, Nursing and Management, No. 79-9, Sha-Luen-Hu, Xi-Zhou Li, Hou-Loung Town, Miaoli 35664, Taiwan; d9700101@gmail.com; 2 Department of Pediatrics, Wei Gong Memorial Hospital, Miaoli 35159, Taiwan; 045652@tool.caaumed.org.tw (S.-H.K.);; 3 School of Medicine, College of Medicine, Fu Jen Catholic University, New Taipei 242062, Taiwan; 4 Division of Pediatric Infectious Diseases, China Medical University Children’s Hospital, China Medical University, Taichung 40447, Taiwan; codan5230@gmail.com; 5 Molecular Infectious Disease Research Center, Chang Gung Memorial Hospital at Linkou, Taoyuan 333423, Taiwan; dinoschen@cgmh.org.tw; 6 Department of Long Term Care, College of Health and Nursing, National Quemoy University, No. 1, University Road, Jinning Township, Kinmen County 892009, Taiwan; ycchang@email.nqu.edu.tw; 7 Department of Healthcare Administration, College of Medical and Health Science, Asia University, Taichung 41354, Taiwan; 8 Division of Neonatology, China Medical University Children’s Hospital, China Medical University, Taichung 40447, Taiwan; littlepo0327@gmail.com; 9 Department of Neurosurgery, Wei Gong Memorial Hospital, Miaoli 35159, Taiwan; 10 Department of Neurosurgery, China Medical University Hospital, China Medical University, Taichung 404327, Taiwan; 11 School of Medicine, College of Medicine, China Medical University, Taichung 404328, Taiwan; 12 Department of Pediatrics, Asia University Hospital, Asia University, Taichung 41300, Taiwan

**Keywords:** route of administration, antibiotics, autism spectrum disorder, attention-deficit/hyperactivity disorder

## Abstract

**Background/Objectives:** Autism spectrum disorder (ASD) and attention-deficit/hyperactivity disorder (ADHD) substantially affect quality of life. Previous studies have not evaluated whether the associations between early-life antibiotic exposure and the risks of ASD and ADHD differ according to the antibiotic agents administered during infancy. **Methods:** This nationwide case–control study used Taiwan’s National Health Insurance Research Database, Birth Reporting Database, and Maternal and Child Health Database. The cohort included 1,693,718 term neonates born between 2004 and 2015 and followed through to 2022. For ASD (*n* = 7265) and ADHD (*n* = 39,056) cases, antibiotic-exposed children were matched 1:1 with unexposed children based on sex, gestational age, birth weight, and birth year. Adjusted hazard ratios (aHRs) and 95% confidence intervals (CIs) were estimated using multivariable Cox proportional hazards models. **Results:** Oral antibiotic exposure was associated with lower ASD but higher ADHD risk, while parenteral exposure was associated with increased risks of both outcomes. Amoxicillin and erythromycin exposure during the first year of life were associated with lower ASD risk, whereas ampicillin exposure at 4–12 months was associated with higher ASD risk (all *p* ≤ 0.033). For ADHD, exposure to amoxicillin/clavulanic acid, cefixime, ampicillin, and ampicillin/sulbactam was associated with increased risk, whereas erythromycin, cephalexin, and sulfamethoxazole-trimethoprim were associated with reduced risk (all *p* < 0.047). **Conclusions:** Associations between early-life antibiotic exposure and the risks of ASD and ADHD varied according to antibiotic agents, route of administration, and timing of exposure. These findings may help explain inconsistencies among previous studies and inform antibiotic prescribing during infancy.

## 1. Introduction

Through modulation of the microbiota–gut–brain axis, early-life exposure to different antibiotic treatments (e.g., different active ingredients and routes of administration) may be differentially associated with the risks of developing autism spectrum disorder (ASD) and attention-deficit/hyperactivity disorder (ADHD), two common neurodevelopmental disorders associated with increased mortality risk [1,2,3,4,5]. ASD and ADHD frequently co-occur, with 20–50% of children with ADHD meeting diagnostic criteria for ASD and 30–80% of children with ASD meeting diagnostic criteria for ADHD. The co-occurrence of these disorders is associated with greater functional impairment and poorer quality of life than either condition alone [5,6].

Antibiotics are widely prescribed during early childhood, with more than two-thirds of children receiving antibiotics before the age of two years and over half receiving at least one course annually [7,8]. Concerns have arisen regarding their widespread and sometimes unnecessary use [9,10]. As the burden of infectious diseases has declined, immune-mediated conditions have become increasingly prevalent [7,11]. Beyond contributing to antimicrobial resistance, early-life antibiotic exposure has been associated with autism spectrum disorder (ASD), attention-deficit/hyperactivity disorder (ADHD), allergic diseases, and other long-term health outcomes, potentially through alterations of the gut microbiome [7,9,12]. The gut microbiome plays a critical role in immune, metabolic, and neurodevelopment and is influenced by delivery mode, infant feeding practices, environmental exposures, and antibiotic use [9,13]. Growing evidence suggests that disruptions of the gut microbiome during critical developmental periods may have lasting consequences for health [9,14]. Kang et al. reported clinical and microbiome-related changes following microbiota transfer therapy in children with ASD [15]. Although the study provides potentially relevant mechanistic context, it does not directly address early-life antibiotic exposure or establish a causal role of microbiome alterations in ASD. Chen et al. provided further evidence that differences in microbiota composition may be associated with ASD while also highlighting the importance of genetic background and shared environmental factors when interpreting microbiome–ASD associations [16]. Therefore, microbiome differences observed in children with ASD should not automatically be interpreted as a primary cause of neurodevelopmental disorders. However, most human evidence linking early-life antibiotic exposure, gut microbiome alterations, ASD, and ADHD remains observational, and the direction of and mechanisms underlying these associations are not fully understood. Children with ASD or ADHD may also differ in diet, gastrointestinal symptoms, medication use, healthcare utilization, and other factors that can influence the gut microbiome.

Previous studies have reported inconsistent associations between early-life antibiotic exposure and the risks of ASD and ADHD [7,9]. However, whether these associations vary according to the pharmaceutical formulation of antibiotics administered during infancy, including individual antibiotic agents and routes of administration, has not been investigated. In this nationwide population-based study, we evaluated these associations according to antibiotic agent and route of administration. To our knowledge, this is the first nationwide study to examine the associations between different antibiotic agents and the subsequent risks of ASD and ADHD.

**Figure 1:**
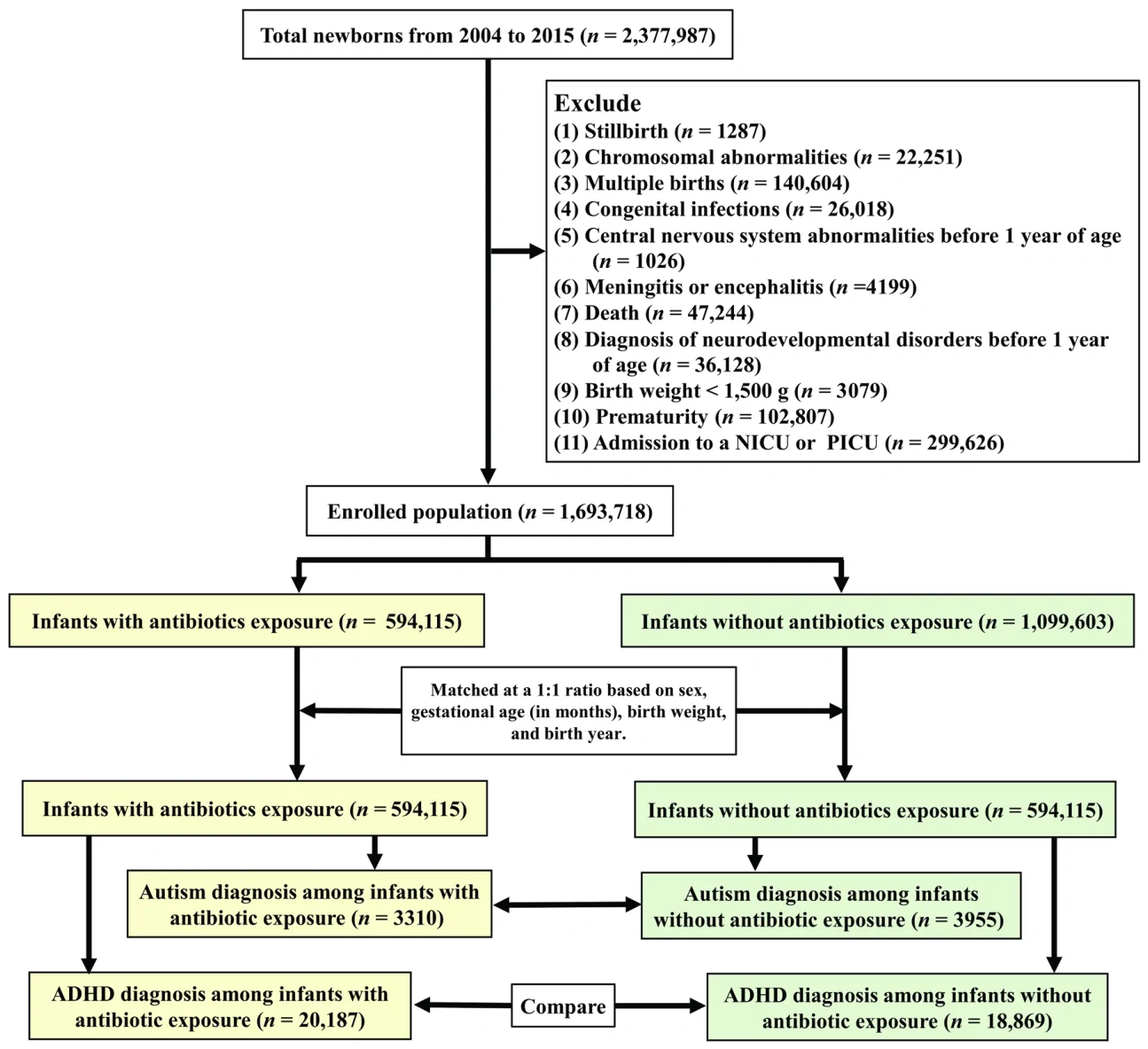
Flowchart of Study Cohort Selection for the Antibiotic Exposure and Non-Exposure Groups Used to Assess the Risks of Autism Spectrum Disorder and Attention-Deficit/Hyperactivity Disorder.

To minimize confounding by severe infections and underlying conditions, we excluded infants admitted to a neonatal or pediatric intensive care unit (NICU or PICU) and those with potentially relevant conditions, including chromosomal abnormalities, congenital infections, central nervous system abnormalities, meningitis or encephalitis, and neurodevelopmental disorders diagnosed before 1 year of age (Figure 1). We also used multivariable Cox proportional hazards models to adjust for parental chronic and psychiatric disorders, neonatal characteristics, and socioeconomic status (SES), thereby reducing confounding from established genetic and environmental risk factors for ASD and ADHD (Table 1) [17,18,19,20].

## 2. Materials and Methods

### 2.1. Data Sources

This nationwide retrospective cohort study used linked data from the Taiwan National Health Insurance Research Database (NHIRD), Birth Reporting Database (BRD), and Maternal and Child Health Database (MCHD) to identify pregnant individuals and their term offspring (≥37 weeks of gestation) born between 2004 and 2015. These nationwide population-based databases contain validated information on demographic characteristics, diagnoses, medication use, perinatal factors, healthcare utilization, and parent–child linkages. De-identified linked records were used to ascertain maternal and neonatal risk factors for autism spectrum disorder (ASD) and attention-deficit/hyperactivity disorder (ADHD).

### 2.2. Ethical Approval

This study was approved by the Institutional Review Board of China Medical University Hospital, Taiwan (CMUH114-REC1-075, CMUH114-REC2-075 and CMUH115-REC1-134). The requirement for informed consent was waived because all datasets were de-identified before analysis. The study was reported in accordance with the Strengthening the Reporting of Observational Studies in Epidemiology (STROBE) guidelines (Appendix Table A1).

### 2.3. Study Population and Design

We included term infants born between 2004 and 2015 and followed them through to 31 December 2022. The study flowchart and exclusion criteria are presented in Figure 1. This study was designed as a retrospective cohort study. For all study participants, the index date (time zero) was defined as 1 year of age (365 days after birth). Participants were followed from the index date until the first occurrence of the study outcome (ASD or ADHD), death, their 18th birthday, or 31 December 2022, whichever came first.

To ensure that the assessment of early-life antibiotic exposure preceded outcome determination, antibiotic exposure was assessed during the first year of life, and children diagnosed with a neurodevelopmental disorder before 1 year of age were excluded. This exclusion criterion is specified in Figure 1 as “Diagnosis of neurodevelopmental disorders before 1 year of age.” Therefore, all ASD and ADHD events included in the analyses occurred after the index date and after completion of the exposure assessment period.

Based on antibiotic prescription records during the first year of life, children were categorized into an unexposed group, consisting of children with no antibiotic prescriptions during the first 12 months of life, and an exposed group, consisting of children with at least one antibiotic prescription during this period. The exposed group was further categorized according to the timing of the first antibiotic exposure into (A) exposure during 0–3 months of age and (B) exposure during 4–12 months of age.

For the primary cohort analyses, propensity score matching (PSM) was used to match children in the antibiotic-exposed and unexposed groups at a 1:1 ratio based on sex, gestational age, birth weight, and birth year, as shown in Table 2. To account for the matched-pair structure in the time-to-event analyses, stratified Cox proportional hazards regression models were used, with each matched pair was treated as a separate stratum. Hazard ratios (HRs) and 95% confidence intervals (CIs) were estimated from these models.

PSM is a statistical technique used in observational studies to balance measured baseline characteristics between exposed and unexposed groups. The propensity score represents the probability of receiving the exposure conditional on observed covariates and is typically estimated using a logistic regression model [21,22,23]. In the present study, the propensity score represented the estimated probability that an infant would receive antibiotic treatment based on the matching variables. By balancing these observed characteristics between the comparison groups, PSM can reduce potential selection bias and improve comparability between exposed and unexposed children.

Demographic characteristics, antibiotic prescription records, diagnostic records, and parental information were obtained from the linked national databases. Because antibiotic exposure was defined during the first year of life and follow-up for ASD and ADHD began at 1 year of age, the exposure period preceded the outcome determination.

### 2.4. Antibiotic Exposure

Antibiotic exposure during the first year of life was identified using Anatomical Therapeutic Chemical (ATC) code J01 (antibacterials for systemic use). Exposure was evaluated during three predefined periods: 0–12 months, 0–3 months, and 4–12 months of age. Overall antibiotic exposure and route of administration (oral versus parenteral) were evaluated as the primary analyses. Analyses according to individual generic antibiotic agents and specific exposure periods were considered secondary or exploratory analyses.

Infants exposed to more than one generic antibiotic during the first year of life were excluded from analyses of individual antibiotic agents to minimize potential exposure misclassification. All antibiotic exposures were assessed before the index date, and therefore, no antibiotic prescriptions occurring after the start of follow-up were included in the exposure definition.

### 2.5. Outcome Determination

The primary outcomes were ASD and ADHD, identified using pooled International Classification of Diseases, Ninth Revision (ICD-9) and Tenth Revision (ICD-10) diagnostic codes. To improve diagnostic validity, cases were defined as children with at least one inpatient diagnosis or three outpatient diagnoses for ASD or ADHD.

### 2.6. Covariates

Potential neonatal and parental confounders were identified from the linked databases and adjusted for in the analyses. Neonatal jaundice was classified as no treatment, simple phototherapy, intensive phototherapy, or exchange transfusion according to procedure codes (57106C, 57117B, and 57105B) (Table 1). Diabetes mellitus requiring insulin treatment was defined as a diagnosis of diabetes mellitus together with prescriptions for insulin (ATC code A10).

Parental allergic disease was defined as asthma, allergic rhinitis, or atopic dermatitis diagnosed in either parent with at least three outpatient visits within any 90-day period. Parental ASD, ADHD, neurodevelopmental disorders, anxiety, depression, and other mental and behavioral disorders were identified using diagnoses recorded within two years before childbirth (Table 1). Family income was categorized into birth year-specific quartiles according to insured income at delivery. Urbanization level was determined according to the mother’s residential area and classified using the method proposed by Liu et al. [24].

### 2.7. Statistical Analysis

Statistical analyses were performed using SAS version 9.4 (SAS Institute, Cary, NC, USA). Two-sided *p* values < 0.05 were considered statistically significant. Baseline characteristics and incidence rates were summarized descriptively. Associations between antibiotic exposure and ASD or ADHD were estimated using multivariable Cox proportional hazards regression models and reported as adjusted hazard ratios (aHRs) with 95% confidence intervals (CIs), adjusting for neonatal, parental, and area-level socioeconomic factors. Analyses of overall antibiotic exposure and route of administration (oral versus parenteral) were considered the primary analyses, whereas analyses according to individual antibiotic agents and specific exposure periods were considered secondary or exploratory analyses. Two sensitivity analyses were conducted to reduce confounding by infection severity and type: (1) analyses restricted to children with respiratory tract infections before 1 year of age and no other documented infections, and (2) analyses restricted to children without prolonged hospitalization during the first year of life.

## 3. Results

### 3.1. Study Population and Baseline Characteristics

Among 2,377,987 neonates born between 2004 and 2015, 1,693,718 term infants were eligible after applying the exclusion criteria. Of these, 594,115 (35.1%) were exposed to at least one systemic antibiotic during the first year of life.

For the case–control analyses, 7265 children with ASD and 39,056 with ADHD were identified. Antibiotic-exposed children were matched 1:1 with unexposed children based on sex, gestational age, birth weight, and birth year (Figure 1). As expected, no significant differences were observed between the matched groups for these variables (all *p* = 1.0) (Table 2).

Among all matched participants, 52.12% were male. The mean gestational age was 38.64 ± 1.06 weeks, the mean birth weight was 3148 ± 375 g, and 33.29% were delivered by cesarean section.

### 3.2. Associations Between Antibiotic Exposure and the Risks of ASD and ADHD

**Figure 2:**
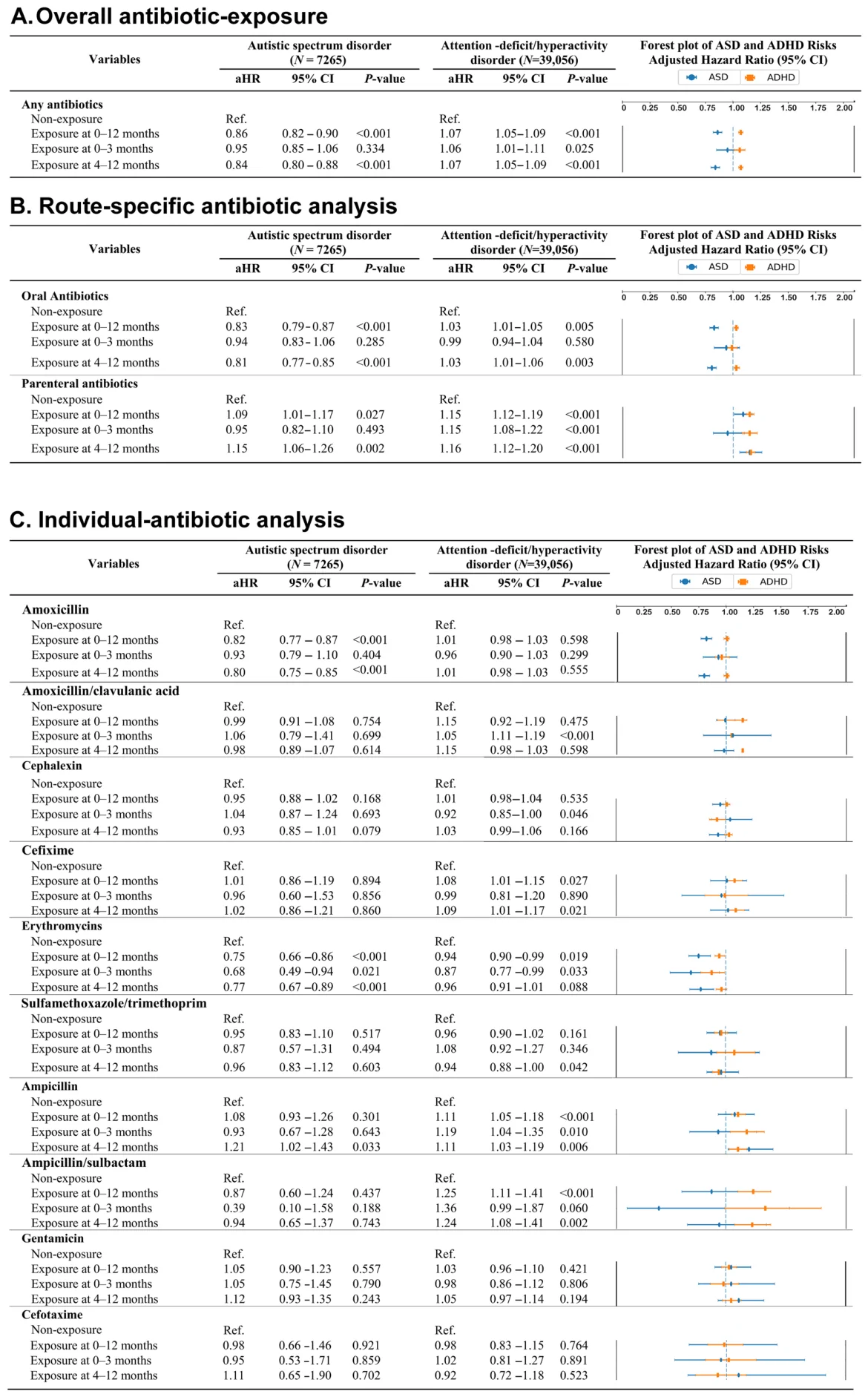
Cox proportional hazards regression analyses and forest plots showing the associations between antibiotic exposure and the risks of autism spectrum disorder and attention-deficit/hyperactivity disorder. Abbreviations: Ref., reference group; aHR, adjusted hazard ratio; CI, confidence interval.

The associations between antibiotic exposure and the risks of ASD and ADHD are summarized in Figure 2. The numbers of exposed children and outcome events for the main individual-antibiotic analyses, together with the corresponding absolute incidence rates, are presented in Appendix Table A2.

Overall, antibiotic exposure during 4–12 months or throughout the first year of life (0–12 months) was associated with a reduced risk of ASD (both *p* < 0.001). In contrast, antibiotic exposure during 0–3, 4–12, or 0–12 months was associated with an increased risk of ADHD (all *p* < 0.026).

During the first year of life, 538,908 infants received oral antibiotics and 119,813 received parenteral antibiotics. Oral antibiotic exposure during 4–12 or 0–12 months was associated with a lower risk of ASD (both *p* < 0.001) but a higher risk of ADHD (both *p* < 0.006). In contrast, parenteral antibiotic exposure was associated with increased risks of both ASD (both *p* < 0.028) and ADHD (both *p* < 0.001).

Among 97,845 infants exposed during the first 3 months of life and 496,270 exposed during 4–12 months, early antibiotic exposure was generally not associated with ASD or ADHD. The only exception was parenteral antibiotic exposure during 0–3 months, which was associated with an increased risk of ADHD (*p* < 0.001).

Many individual antibiotic agents, including cefadroxil, clarithromycin, azithromycin, cefazolin, gentamicin, cefotaxime, ceftazidime, and ceftriaxone, were not significantly associated with ASD or ADHD. Amoxicillin and erythromycin exposure during 4–12 or 0–12 months was associated with a reduced risk of ASD, whereas erythromycin exposure during 0–3 months was also associated with lower ASD risk. Conversely, ampicillin exposure during 4–12 months was associated with an increased risk of ASD (*p* = 0.033). 

For ADHD, exposure to amoxicillin–clavulanic acid, cefixime, ampicillin, and ampicillin–sulbactam during 4–12 or 0–12 months was associated with increased risk (all *p* < 0.028). Ampicillin exposure during 0–3 months, erythromycin exposure during 0–3 or 0–12 months, and vancomycin exposure during 0–12 months were also associated with increased ADHD risk (all *p* < 0.034). In contrast, cephalexin exposure during 0–3 months and sulfamethoxazole–trimethoprim exposure during 4–12 months were associated with reduced ADHD risk (both *p* < 0.047). These findings should be interpreted cautiously, particularly when effect estimates are small or *p* values are close to the conventional significance threshold of 0.05.

### 3.3. Associations of Neonatal and Parental Factors with ASD and ADHD

**Figure 3:**
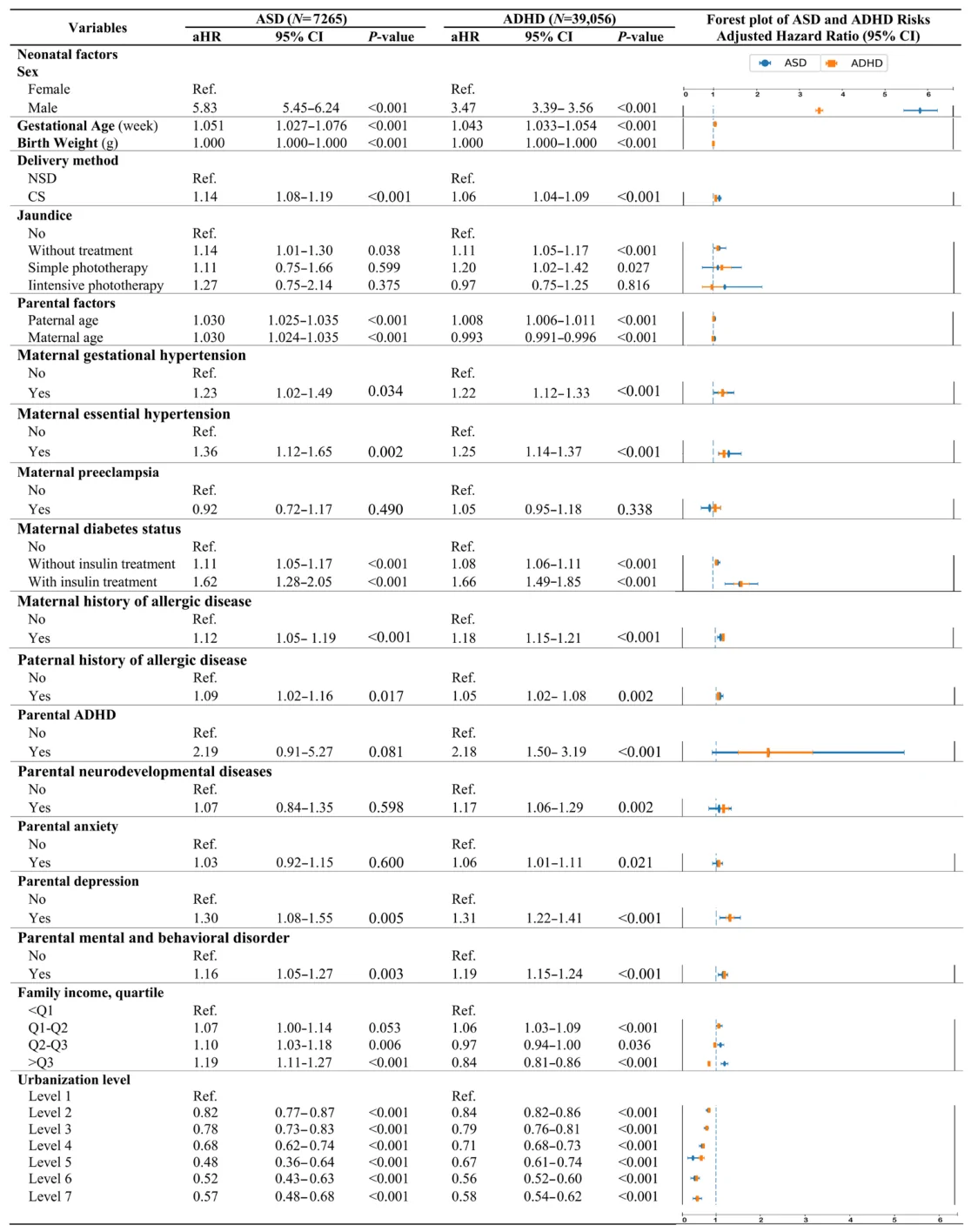
Cox Proportional Hazards Regression Analysis and Forest Plot Showing the Associations Between Neonatal and Parental Factors and the Risks of Autism Spectrum Disorder and Attention-Deficit/Hyperactivity Disorder. Abbreviations: Ref., reference group; aHR, adjusted hazard ratio; 95% CI, confidence interval; NSD, normal spontaneous delivery; CS, cesarean section; ADHD, attention-deficit/hyperactivity disorder; Q1, first quartile; Q2, second quartile; Q3, third quartile.

The associations between neonatal and parental characteristics and the risks of ASD and ADHD are shown in Figure 3.

Among term infants, lower gestational age, lower birth weight, male sex, cesarean delivery, untreated neonatal jaundice, advanced paternal age, maternal gestational or chronic hypertension, maternal diabetes mellitus, parental allergic diseases, and parental depression or other mental and behavioral disorders were independently associated with increased risks of both ASD and ADHD (all *p* < 0.001).

Neonatal jaundice requiring phototherapy and parental ADHD, neurodevelopmental disorders, anxiety, or depression were additionally associated with an increased risk of ADHD (all *p* < 0.041).

Compared with children from the lowest family income quartile (Q1), those from the highest income quartile (Q4) had a higher risk of ASD (aHR = 1.19) but a lower risk of ADHD (aHR = 0.84). Lower urbanization levels (levels 5–7) were associated with lower risks of both ASD (aHR, 0.48–0.57) and ADHD (aHR, 0.56–0.67) compared with the highest urbanization level (level 1).

### 3.4. Sensitivity Analyses

Sensitivity analyses restricted to children with respiratory tract infections during the first year of life and no other documented infections, as well as those without prolonged hospitalization before 1 year of age, yielded results consistent with the primary analyses (Appendix Table A3), supporting the robustness of the observed associations.

## 4. Discussion

To our knowledge, this is the first study to investigate the associations between antibiotic route of administration (oral versus parenteral) and the risks of ASD and ADHD. Although previous studies have evaluated antibiotic classes, broad- versus narrow-spectrum antibiotics, or overall antibiotic exposure [7,9,25], none have systematically examined individual antibiotics by generic name in relation to these neurodevelopmental outcomes. Our findings suggest that both the route of administration and specific antibiotic agents are associated with the subsequent risks of ASD and ADHD. Differences in antibiotic prescribing practices, healthcare systems, and commonly used agents across countries may partly explain the inconsistent findings reported in previous studies [7,9,26]. 

A population-based cohort study from Manitoba, Canada, reported a lower risk of ASD following antibiotic exposure during the first year of life (adjusted hazard ratio [aHR], 0.91; 95% confidence interval [CI], 0.84–0.99) [27]. Similarly, we observed lower ASD risk following antibiotic exposure during 4–12 months and 0–12 months of age. A Minnesota cohort found an increased risk of ADHD but not ASD after early-life antibiotic exposure [9], whereas a Korean cohort reported no overall association with ASD but increased risk among infants exposed very early or for prolonged durations [25]. Together, these findings suggest that the timing, duration, and type of antibiotic exposure may influence neurodevelopmental outcomes.

Several other population-based studies have also reported inconsistent findings. A Swedish population-based cohort study reported stronger associations between early-life antibiotic exposure and both ASD and ADHD [28]. Specifically, early antibiotic exposure was associated with ASD (OR = 1.46, 95% CI: 1.38–1.55) and ADHD (OR = 1.90, 95% CI: 1.80–2.00) [28]. However, the authors noted that confounding by indication, genetic susceptibility, and other unmeasured factors could not be excluded.

A recent meta-analysis of 10 studies published between 2016 and 2020 found that early-life antibiotic exposure was associated with modestly increased risks of ASD (OR = 1.13, 95% CI: 1.07–1.21) and ADHD (OR = 1.18, 95% CI: 1.10–1.27) [29]. However, these associations were no longer significant when sibling-matched studies were pooled (ASD: OR = 1.04, 95% CI: 0.97–1.11; ADHD: OR = 0.98, 95% CI: 0.94–1.02), suggesting that familial factors may partly account for the observed associations [27]. Because only a limited number of sibling-matched studies were included, the authors emphasized that these findings should be interpreted cautiously [29].

Similarly, a Danish population-based study of 671,606 children born between 1997 and 2010 found no evidence supporting a causal association between antibiotic treatment during infancy and autism [30].

Several studies with findings consistent with ours have reported an increased risk of ADHD following antibiotic exposure. A population-based study from Taiwan found that the risk of ADHD was highest among children exposed to antibiotics before 2 years of age, regardless of exposure duration. Among children older than 2 years, increased ADHD risk was associated with prolonged antibiotic exposure [31]. In addition, a Finnish population-based study that included 990,098 live births between 1996 and 2012 found a modest association between in utero and early-life antibiotic exposure and subsequent ADHD during childhood [32]. A New Zealand cohort study involving 342 children also reported a positive association between antibiotic exposure at different time points during the first 2 years of life and ADHD at 11 years of age [33].

Nevertheless, several studies have reported no significant association between antibiotic exposure and ADHD. A retrospective cohort study in the United Kingdom involving 1,091,449 children born between 1987 and 2020 found that antibiotic exposure before 2 years of age was not associated with ASD or ADHD [34]. Similarly, a Danish population-based study including 671,592 children born between 1997 and 2010 found no association between antibiotic treatment during the first 2 years of life and the risk of ADHD [35]. In a Canadian study conducted between 1998 and 2017, antibiotic exposure was not associated with ADHD risk in either the matched cohort (HR = 1.02, 95% CI: 0.97–1.08) or the sibling cohort (HR = 0.96, 95% CI: 0.89–1.03) [36].

These discrepancies may be attributable to differences in study design, study populations, study periods, sample sizes, exposure definitions, outcome determination, and analytical approaches. In particular, variations in methods of confounding adjustment, antibiotic prescribing patterns across healthcare systems, timing and duration of antibiotic exposure, and classification of antibiotic agents may have contributed to the divergent findings across studies.

Unlike previous studies that primarily evaluated antibiotic classes, our study examined individual antibiotics by generic name. Amoxicillin and erythromycin exposures during infancy were associated with lower ASD risk, consistent with previous reports showing protective associations for penicillins and macrolides [9,27]. In contrast, ampicillin exposure during 4–12 months was associated with increased ASD risk. To our knowledge, this is the first study to report an association between ampicillin, which is commonly administered intravenously during infancy, and subsequent ASD risk.

For ADHD, exposure to amoxicillin–clavulanic acid, ampicillin, ampicillin–sulbactam, and cefixime was associated with increased risk, whereas cephalexin and sulfamethoxazole–trimethoprim were associated with reduced risk. A previous US study similarly reported an increased ADHD risk associated with penicillin exposure among girls [9]. These findings suggest that neurodevelopmental effects may differ substantially among individual antibiotics rather than across antibiotic classes alone.

An additional novel finding was that parenteral antibiotic exposure was associated with increased risks of both ASD and ADHD, whereas oral antibiotic exposure was associated with lower ASD risk but higher ADHD risk. One possible explanation is that parenteral antibiotics are generally prescribed for more severe infections and may result in greater disruption of the developing gut microbiome than oral antibiotics. Alternatively, route of administration may serve as a surrogate marker for infection severity or hospitalization, although our sensitivity analyses restricted to children without prolonged hospitalization yielded similar results.

Kang et al. reported clinical and microbiome-related changes following microbiota transfer therapy in children with ASD [15]. However, this study had important limitations, including a small sample size, an open-label design, the lack of a randomized placebo-controlled comparison, and the potential for placebo, behavioral, and other nonspecific effects. Thus, although the study provides potentially relevant mechanistic context, it does not directly address early-life antibiotic exposure or establish a causal role for microbiome alterations in ASD. Chen et al. provided further evidence that differences in microbiota composition may be associated with ASD while also highlighting the importance of genetic background and shared environmental factors when interpreting microbiome–ASD associations [16]. Therefore, microbiome differences observed in children with ASD should not automatically be interpreted as a primary cause of neurodevelopmental disorders.

Although the microbiota–gut–brain axis represents a biologically plausible pathway through which early-life antibiotic exposure could potentially influence neurodevelopment, our observational database study did not assess gut microbiota, microbial metabolites, immune markers, or other mechanistic intermediates. Therefore, our findings cannot establish whether microbiome alterations mediate the observed associations between antibiotic exposure and ASD or ADHD. The microbiota–gut–brain axis should therefore be considered a potential explanatory mechanism that requires further investigation and confirmation through prospective longitudinal, mechanistic, and intervention studies.

Consistent with previous studies, lower gestational age, male sex, cesarean delivery, neonatal jaundice, maternal hypertension or diabetes, parental allergic diseases, and parental psychiatric disorders were associated with increased risks of ASD and/or ADHD [17,37,38,39,40,41,42,43,44]. Higher family income was associated with increased ASD but reduced ADHD risk, whereas lower urbanization levels were associated with lower risks of both disorders, findings that agree with previous reports demonstrating socioeconomic and geographic disparities in neurodevelopmental diagnoses [15,45,46,47,48].

This nationwide population-based study included more than 1.69 million term infants with long-term follow-up, providing substantial statistical power and minimizing selection bias. Comprehensive prescription records enabled analyses according to individual antibiotic agents and routes of administration, extending beyond previous studies that focused on antibiotic classes. We further reduced confounding by excluding infants with meningitis, encephalitis, or neonatal/pediatric intensive care unit admission and adjusted for numerous neonatal, parental, and socioeconomic factors.

Several limitations should be considered. First, because this was an observational study, causal relationships cannot be established, and residual confounding cannot be completely excluded. Although we excluded children admitted to the PICU or NICU and those with potentially relevant underlying conditions, including chromosomal abnormalities, congenital infections, central nervous system abnormalities, meningitis or encephalitis, and diagnoses of neurodevelopmental disorders before 1 year of age, and further adjusted for parental chronic and psychiatric disorders, neonatal characteristics, and socioeconomic status (SES) in the multivariable Cox proportional hazards models, some residual differences in clinical circumstances may remain. In particular, differences in infection severity, healthcare-seeking behavior, and healthcare setting between children receiving oral versus parenteral antibiotics may have contributed to the observed associations. Therefore, these findings should be interpreted as associations rather than evidence of causality. 

Second, important variables such as breastfeeding, maternal lifestyle, environmental exposures, infection severity, and gut microbiome composition were unavailable. Third, antibiotic exposure was determined from prescription claims rather than confirmed medication use. Fourth, although validated diagnostic criteria were applied, ASD and ADHD were identified using administrative databases and some misclassification is possible.

Fifth, sibling-matched analyses were not performed in the present study. To minimize potential confounding related to shared intrauterine environments and the high prevalence of gestational complications, multiple births were explicitly excluded. Furthermore, sibling-matched designs involving siblings born in different years may remain susceptible to time-varying confounding, including changes in clinical guidelines and antibiotic prescribing practices. Therefore, we prioritized a same-birth-year matched cohort design to control for period-specific environmental, diagnostic, and prescribing trends. Although within-family comparisons could potentially reduce residual confounding related to shared genetic, familial, socioeconomic, and environmental factors, they may also reduce statistical power and generalizability and introduce additional sources of bias. Therefore, sibling analyses should be considered complementary rather than definitive evidence of causality. In addition, sibling relationships were not directly available in the analytical dataset used for this study. A robust sibling analysis would require additional family-level data linkage, reconstruction and validation of eligible sibling pairs, and a specifically designed within-family analytical framework. Future studies using a dedicated family-linked cohort are warranted to determine whether the observed associations remain consistent after accounting for shared familial factors.

Sixth, the number of antibiotic courses, treatment duration, and cumulative antibiotic exposure could not be reliably determined from the Taiwan National Health Insurance Research Database (NHIRD), Birth Reporting Database (BRD), or Maternal and Child Health Database (MCHD) available for this study. Therefore, we were unable to assess cumulative antibiotic exposure or evaluate potential dose- or duration-response relationships. 

Seventh, given the observational study design and the heterogeneous associations observed across antibiotic classes, routes of administration, and exposure windows, the findings should be interpreted as associations rather than causal effects. In particular, the apparently reduced risk of ASD associated with some antibiotics should be interpreted cautiously, as these findings may reflect residual confounding, differences in clinical indications, or other unmeasured factors rather than a protective effect of antibiotic exposure.

Finally, although each individual analysis involved a two-group comparison, the multiple analyses conducted across antibiotic agents, exposure periods, and routes of administration may have increased the risk of type I error. No formal multiple-testing correction, such as the false discovery rate, was applied because these analyses were considered secondary and exploratory. Therefore, the agent-specific findings, particularly those with borderline *p* values, should be interpreted cautiously and regarded as hypothesis-generating rather than definitive.

## 5. Conclusions

In this nationwide population-based study of term neonates, we found that different antibiotic agents and routes of administration were differentially associated with the risks of developing ASD and ADHD. Oral antibiotic exposure was associated with a lower risk of ASD but a higher risk of ADHD, whereas parenteral antibiotic exposure was associated with increased risks of both outcomes. Among individual antibiotic agents, amoxicillin and erythromycin exposure during the first year of life was associated with a reduced risk of ASD, whereas ampicillin exposure during 4–12 months was associated with an increased risk of ASD. For ADHD, exposure to amoxicillin/clavulanic acid, cefixime, ampicillin, and ampicillin/sulbactam was associated with an increased risk, whereas erythromycin, cephalexin, and sulfamethoxazole–trimethoprim were associated with a reduced risk. However, these findings represent associations rather than causal effects, particularly given the heterogeneous findings across antibiotic classes, routes of administration, and exposure windows.

To our knowledge, this is the first nationwide population-based study to evaluate the associations of individual antibiotic agents and routes of administration with the subsequent risks of ASD and ADHD. These findings suggest that the neurodevelopmental associations of early-life antibiotic exposure may vary according to the specific antibiotic agent, route of administration, and timing of exposure. Differences in prescribing practices across healthcare systems may partly explain the inconsistent findings reported in previous studies. These results may help inform more judicious antibiotic prescribing during infancy and guide future studies investigating the potential neurodevelopmental effects of individual antibiotic agents.

## Figures and Tables

**Table 1:** Diagnoses of Autism Spectrum Disorder, Attention-Deficit/Hyperactivity Disorder, and Associated Risk Factors.

Outcomes or Risk Factors	ICD-9-CM Codes (2004–2015)	ICD-10-CM Codes (2016–2022)
**Autistic spectrum disorder (ASD)**	299.0, 299.00, 299.01, 299.80, 299.90	F84.0, F84.5, F84.8, F84.9
**Attention deficit hyperactivity disorder (ADHD)**	314.00, 314.01, 314.1, 314.9	F90.0, F90.1, F90.2, F90.8, F90.9
**Neonatal risk factors**		
Delivery by Cesarean section	669.70, 669.71, V30.01, V31.01, V32.01, V33.01, V34.01, V35.01, V36.01, V37.01, V39.01	O82, Z38.01, Z38.31, Z38.62, Z38.64, Z38.66, Z38.69, DRG 765, DRG 766
Jaundice	774.xx	P55–P59, R17
**Neurodevelopmental diseases**		
Cerebral palsy	343.xx	G80.x
Language developmental disorder	315.31, 315.39	F80.x
Hearing impairment	389.xx	H90.x, H91.x
Blindness, bilateral	369.00, 369.3, 369.4, 369.9	H54.x
**Maternal risk factors**		
Gestational [pregnancy-induced] hypertension	642.30–642.34, 642.40–642.44	O13.x
Essential hypertension	401.x	I10
Preeclampsia	642.40–642.74	O14.x
Maternal diabetes mellitus	250.xx, 648.0x	E08–E13, O24.x
Allergic diseases (asthma, allergic rhinitis, and atopic dermatitis)	493.xx, 477.xx, 691.xx	J45.x, J30.x, L20.x
Anxiety	300.0, 300.2, 300.3	F40–F42, F93.0–F93.2
Depression	296.2, 296.3, 300.4, 311	F32–F34.1, F38.1
Mental and behavioral disorders	290–319	F01-F99

**Table 2:** Baseline characteristics of participants matched at a 1:1 ratio in this case–control study.

Variable	Antibiotic Exposure Group (*N* = 594,115)	Antibiotic Non-Exposure Group (*N* = 594,115)	*p* Value
**Sex**			1.000
Male	309,677 (52.12)	309,677 (52.12)	
Female	284,438 (47.88)	284,438 (47.88)	
**Gestational age (week)**			1.000
37	89,862 (15.13)	89,862 (15.13)	
38	189,875 (31.96)	189,875 (31.96)	
39	182,312 (30.69)	182,312 (30.69)	
40+	132,066 (22.23)	132,066 (22.23)	
Mean ± SD	38.64 ± 1.06	38.64 ± 1.06	
**Birth weight (g)**			1.000
3380+ (>Q3)	153,469 (25.83)	153,469 (25.83)	
3120~3379 (Q2–Q3)	152,060 (25.59)	152,060 (25.59)	
2900~3119 (Q1–Q2)	145,208 (24.44)	145,208 (24.44)	
1500~2899 (<Q1)	143,378 (24.13)	143,378 (24.13)	
Mean ± SD	3147.46 ± 374.10	3147.46 ± 374.10	
**Birth Year**			1.000
2004	59,937 (10.09)	59,937 (10.09)	
2005	61,398 (10.33)	61,398 (10.33)	
2006	55,409 (9.33)	55,409 (9.33)	
2007	52,784 (8.88)	52,784 (8.88)	
2008	49,690 (8.36)	49,690 (8.36)	
2009	48,415 (8.15)	48,415 (8.15)	
2010	39,627 (6.67)	39,627 (6.67)	
2011	45,976 (7.74)	45,976 (7.74)	
2012	50,512 (8.50)	50,512 (8.50)	
2013	42,453 (7.15)	42,453 (7.15)	
2014	41,379 (6.96)	41,379 (6.96)	
2015	46,535 (7.83)	46,535 (7.83)	

*N*, total number, presented as *n* (%).

## Data Availability

The data analyzed in this study were provided from the Health and Welfare Data Science Center (HWDSC) affiliated with the Ministry of Health and Welfare in Taiwan. Due to legal restrictions imposed by the Taiwan government related to the Personal Information Protection Act, the databases cannot be made publicly available. Requests for access to these data should be sent as a formal proposal to the HWDSC in Taiwan (https://dep.mohw.gov.tw/dos/cp-5119-59201-113.html) (accessed on 1 January 2026).

## Appendix A

**Table A1:** STROBE checklist for this study.

Item	Recommendation	Location in Manuscript
**Title and Abstract**		
1a	Indicate the study design with a commonly used term in the title or abstract (e.g., nationwide population-based cohort study).	Title/Abstract
1b	Provide an informative and balanced summary of objectives, methods, results, and conclusions.	Abstract
**Introduction**		
2	Explain the scientific background and rationale.	Introduction
3	State specific objectives and hypotheses.	Introduction
**Methods**		
4	Present key elements of study design early in the paper.	Methods
5	Describe setting, locations, relevant dates, recruitment period, exposure period, and follow-up.	Methods
6a	Describe eligibility criteria, sources, and methods of participant selection; methods of follow-up.	Methods
6b	For matched studies, describe matching criteria.	Methods
7	Clearly define outcomes, exposures, confounders, and effect modifiers; provide diagnostic criteria.	Methods
8*	Describe data sources and measurement methods for each variable.	Methods
9	Describe efforts to address potential bias.	Methods
10	Explain how study size was determined.	Methods
11	Explain handling of quantitative variables and categorization.	Statistical Analysis
12a	Describe statistical methods, including confounder adjustment.	Statistical Analysis
12b	Describe subgroup analyses and interaction analyses.	Statistical Analysis
12c	Explain handling of missing data.	Statistical Analysis/Methods
12d	Explain handling of loss to follow-up.	Methods
12e	Describe sensitivity analyses.	Statistical Analysis/Results
**Results**		
13a	Report numbers at each stage (eligible, included, followed, analyzed).	Results/Flowchart
13b	Give reasons for non-participation or exclusions.	Flowchart/Results
13c	Consider a flow diagram.	Figure 1
14a	Describe participant characteristics and exposures.	Table 1
14b	Report missing data for each variable.	Results
14c	Summarize follow-up time.	Results
15	Report numbers of outcome events or summary measures over time.	Results
16a	Report unadjusted and adjusted estimates with precision (95% CI); specify confounders included.	Figure 2 and Figure 3
16b	Report category boundaries for categorized continuous variables.	Methods/Tables
16c	Consider translating relative risks into absolute risks.	Figure 2 and Figure 3
17	Report subgroup analyses, interaction analyses, and sensitivity analyses.	Results
**Discussion**		
18	Summarize key findings in relation to objectives.	Discussion
19	Discuss limitations, including potential bias and imprecision.	Strengths and Limitations
20	Provide an overall interpretation considering objectives, limitations, multiplicity of analyses, and previous evidence.	Discussion
21	Discuss generalizability of the findings.	Discussion
**Other Information**		
22	Report funding source and role of funders.	Funding Statement

**Table A2:** Cox proportional hazards regression analysis of the association between antibiotic exposure and the risk of autism spectrum disorder and attention disorder.

	Number of Children	Autistic Spectrum Disorder (n = 7265)					Attention-Deficit/Hyperactivity Disorder (n = 39,056)				
Variable		Events	% ^a^	aHR	95% CI	*p*-Value	Events	%	aHR	95% CI	*p*-Value
**Overall antibiotic exposure**											
**Any antibiotic**											
No exposure	594,115	3955	0.67	Ref.			18,869	3.18	Ref.		
Exposure at 0–12 months	594,115	3310	0.56	0.86	0.82–0.90	<0.001	20,187	3.40	1.07	1.05–1.09	<0.001
Exposure at 0–3 months	97,845	609	0.62	0.95	0.85–1.06	0.334	3376	3.45	1.06	1.01–1.11	0.025
Exposure at 4–12 months	496,270	2701	0.54	0.84	0.80–0.88	<0.001	16,811	3.39	1.07	1.05–1.09	<0.001
**Route-specific analysis**											
**Oral Antibiotics**											
No exposure	749,322	4895	0.65	Ref.			24,396	3.26	Ref.		
Exposure at 0–12 months	438,908	2370	0.54	0.83	0.79 0.87	<0.001	14,660	3.34	1.03	1.01–1.05	0.005
Exposure at 0–3 months	58,101	349	0.60	0.94	0.83–1.06	0.285	1900	3.27	0.99	0.94–1.04	0.580
Exposure at 4–12 months	380,807	2018	0.53	0.81	0.77–0.85	<0.001	12,760	3.35	1.03	1.01–1.06	0.003
**Parenteral antibiotics**											
No exposure	1,068,417	6478	0.61	Ref.			34,453	3.22	Ref.		
Exposure at 0–12 months	119,813	787	0.66	1.09	1.01–1.17	0.027	4603	3.84	1.15	1.12–1.19	<0.001
Exposure at 0–3 months	34,884	231	0.66	0.95	0.82–1.10	0.493	1359	3.90	1.15	1.08–1.22	<0.001
Exposure at 4–12 months	84,929	556	0.65	1.15	1.06–1.26	0.002	3244	3.82	1.16	1.12–1.20	<0.001
**Individual antibiotic analysis**											
**Amoxicillin**											
No exposure	1,068,076	6652	0.62	Ref.			35,067	3.28	Ref.		
Exposure at 0–12 months	120,154	613	0.51	0.82	0.77–0.87	<0.001	3989	3.32	1.01	0.98–1.03	0.598
Exposure at 0–3 months	13,403	79	0.59	0.93	0.79–1.10	0.404	434	3.24	0.96	0.90–1.03	0.299
Exposure at 4–12 months	106,751	534	0.50	0.80	0.75–0.85	<0.001	3555	3.33	1.01	0.98–1.03	0.555
**Amoxicillin/clavulanic acid**											
No exposure	1,091,741	6680	0.61	Ref.			35,379	3.24	Ref.	1.11–1.19	<0.001
Exposure at 0–12 months	96,489	585	0.61	0.99	0.91–1.08	0.754	3677	3.81	1.15	0.92–1.19	0.475
Exposure at 0–3 months	7192	49	0.68	1.06	0.79–1.41	0.699	260	3.62	1.05	1.11–1.19	<0.001
Exposure at 4–12 months	89,297	536	0.60	0.98	0.89–1.07	0.614	3417	3.83	1.15	0.98–1.03	0.598
**Cephalexin**											
No exposure	1,108,832	6874	0.62	Ref.			36,356	3.28	Ref.		
Exposure at 0–12 months	79,398	461	0.58	0.95	0.88–1.02	0.168	2700	3.40	1.01	0.98–1.04	0.535
Exposure at 0–3 months	12,375	85	0.69	1.04	0.87–1.24	0.693	412	3.33	0.92	0.85–1.00	0.046
Exposure at 4–12 months	67,023	376	0.56	0.93	0.85–1.01	0.079	2288	3.41	1.03	0.99–1.06	0.166
**Cefixime**											
No exposure	1,163,046	7111	0.61	Ref.			38,142	3.28	Ref.		
Exposure at 0–12 months	25,184	154	0.61	1.01	0.86–1.19	0.894	914	3.63	1.08	1.01–1.15	0.027
Exposure at 0–3 months	2936	18	0.61	0.96	0.60–1.53	0.856	100	3.41	0.99	0.81–1.20	0.890
Exposure at 4–12 months	22,248	136	0.61	1.02	0.86–1.21	0.860	814	3.66	1.09	1.01–1.17	0.021
**Erythromycins**											
No exposure	1,135,326	7033	0.62	Ref.			37,383	3.29	Ref.		
Exposure at 0–12 months	52,904	232	0.44	0.75	0.66–0.86	<0.001	1673	3.16	0.94	0.90–0.99	0.019
Exposure at 0–3 months	8595	36	0.42	0.68	0.49–0.94	0.021	251	2.92	0.87	0.77–0.99	0.033
Exposure at 4–12 months	44,309	196	0.44	0.77	0.67–0.89	<0.001	1422	3.21	0.96	0.91–1.01	0.088
**Sulfamethoxazole/trimethoprim**											
No exposure	1,152,258	7064	0.61	Ref.			37,891	3.29	Ref.		
Exposure at 0–12 months	35,972	201	0.56	0.95	0.83–1.10	0.517	1165	3.24	0.96	0.90–1.02	0.161
Exposure at 0–3 months	4005	23	0.57	0.87	0.57–1.31	0.494	155	3.87	1.08	0.92–1.27	0.346
Exposure at 4–12 months	31,967	178	0.56	0.96	0.83–1.12	0.603	1010	3.16	0.94	0.88–1.00	0.042
**Ampicillin**											
No exposure	1,137,998	6926	0.61	Ref.			37,094	3.26	Ref.		
Exposure at 0–12 months	50,232	339	0.67	1.08	0.93–1.26	0.301	1962	3.91	1.11	1.05–1.18	<0.001
Exposure at 0–3 months	23,955	153	0.64	0.93	0.67–1.28	0.643	952	3.97	1.19	1.04–1.35	0.010
Exposure at 4–12 months	26,277	186	0.71	1.21	1.02–1.43	0.033	1010	3.84	1.11	1.03–1.19	0.006
**Ampicillin/sulbactam**											
No exposure	1,182,517	7235	0.61	Ref.			38,792	3.28	Ref.		
Exposure at 0–12 months	5713	30	0.53	0.87	0.60–1.24	0.437	264	4.62	1.25	1.11–1.41	<0.001
Exposure at 0–3 months	805	2	0.25	0.39	0.10–1.58	0.188	39	4.84	1.36	0.99–1.87	0.060
Exposure at 4–12 months	4908	28	0.57	0.94	0.65–1.37	0.743	225	4.58	1.24	1.08–1.41	0.002
**Gentamicin**											
No exposure	1,135,453	6906	0.61	Ref.			37,072	3.26	Ref.		
Exposure at 0–12 months	52,777	359	0.68	1.05	0.90–1.23	0.557	1984	3.76	1.03	0.96–1.10	0.421
Exposure at 0–3 months	22,341	145	0.65	1.05	0.75–1.45	0.790	865	3.87	0.98	0.86–1.12	0.806
Exposure at 4–12 months	30,436	213	0.70	1.12	0.93–1.35	0.243	1119	3.68	1.05	0.97–1.14	0.194
**Cefotaxime**											
No exposure	1,184,500	7239	0.61	Ref.			38,907	3.28	Ref.		
Exposure at 0–12 months	3730	26	0.70	0.98	0.66–1.46	0.921	149	3.99	0.98	0.83–1.15	0.764
Exposure at 0–3 months	2030	12	0.59	0.95	0.53–1.71	0.859	85	4.19	1.02	0.81–1.27	0.891
Exposure at 4–12 months	1700	14	0.82	1.11	0.65–1.90	0.702	64	3.76	0.92	0.72–1.18	0.523

Abbreviations: Ref., reference group; aHR, adjusted hazard ratio; 95% CI, 95% confidence interval. ^a^ % = (number of events/number of exposed or unexposed children) × 100%.

**Table A3:** Sensitivity Analyses for Associations Between any Antibiotic Prescription and Incident Chronic Pediatric Conditions.

Outcome	Autism Spectrum Disorder aHR (95% CI) ^a^			Attention-Deficit/Hyperactivity Disorder aHR (95% CI) ^a^		
	Primary Analysis	RI Only ^b^	No Prolonged Admission ^c^	Primary Analysis	RI Only ^b^	No Prolonged Admission ^c^
**Any antibiotics**						
No exposure	Ref.	Ref.	Ref.	Ref.	Ref.	Ref.
Exposure at 0–12 months	0.86 (0.82–0.90)	0.88 (0.81–0.92)	0.90 (0.85–0.96)	1.07 (1.05–1.09)	1.05 (1.03–1.07)	1.08 (1.06–1.09)
Exposure at 0–3 months	0.95 (0.85–1.06)	0.93 (0.86–1.04)	0.96 (0.88–1.03)	1.06 (1.01–1.11)	1.04 (1.01–1.10)	1.07 (1.01–1.10)
Exposure at 4–12 months	0.84 (0.80–0.88)	0.87 (0.82–0.91)	0.86 (0.81–0.89)	1.07 (1.05–1.09)	1.08 (1.04–1.08)	1.06 (1.03–1.07)
**Oral Antibiotics**						
No exposure	Ref.	Ref.	Ref.	Ref.	Ref.	Ref.
Exposure at 0–12 months	0.83 (0.79–0.87)	0.82 (0.77–0.89)	0.87 (0.78–0.93)	1.03 (1.01–1.05)	1.05 (1.02–1.09)	1.04 (1.01–1.07)
Exposure at 0–3 months	0.94 (0.83–1.06)	0.92 (0.81–1.08)	0.95 (0.84–1.08)	0.99 (0.94–1.04)	0.98 (0.92–1.07)	0.97 (0.92–1.03)
Exposure at 4–12 months	0.81 (0.77–0.85)	0.79 (0.75–0.86)	0.82 (0.76–0.87)	1.03 (1.01–1.06)	1.04 (1.01–1.07)	1.06 (1.02–1.08)
**Parenteral antibiotics**						
No exposure	Ref.	Ref.	Ref.	Ref.	Ref.	Ref.
Exposure at 0–12 months	1.09 (1.01–1.17)	1.11 (1.02–1.18)	1.08 (1.02–1.16)	1.15 (1.12–1.19)	1.16 (1.11–1.17)	1.13 (1.12–1.19)
Exposure at 0–3 months	0.95 (0.82–1.10)	0.96 (0.85–1.07)	0.92 (0.83–1.11)	1.15 (1.08–1.22)	1.13 (1.07–1.21)	1.14 (1.07–1.24)
Exposure at 4–12 months	1.15 (1.06–1.26)	1.16 (1.04–1.27)	1.14 (1.07–1.28)	1.16 (1.12–1.20)	1.15 (1.11–1.26)	1.17 (1.14–1.22)
**Amoxicillin**						
No exposure	Ref.	Ref.	Ref.	Ref.	Ref.	Ref.
Exposure at 0–12 months	0.82 (0.77–0.87)	0.83 (0.76–0.89)	0.87 (0.74–0.91)	1.01 (0.98–1.03)	1.02 (0.99–1.06)	1.03 (0.95–1.07)
Exposure at 0–3 months	0.93 (0.79–1.10)	0.91 (0.76–1.12)	0.92 (0.78–1.11)	0.96 (0.90–1.03)	0.97 (0.91–1.04)	0.96 (0.90–1.03)
Exposure at 4–12 months	0.80 (0.75–0.85)	0.82 (0.73–0.86)	0.81 (0.78–0.84)	1.01 (0.98–1.03)	1.03 (0.96–1.05)	1.06 (0.94–1.05)
**Erythromycin**						
No exposure	Ref.	Ref.	Ref.	Ref.	Ref.	Ref.
Exposure at 0–12 months	0.75 (0.66–0.86)	0.79 (0.68–0.88)	0.81 (0.71–0.90)	0.94 (0.90–0.99)	0.93 (0.87–0.98)	0.95 (0.88–0.99)
Exposure at 0–3 months	0.68 (0.49–0.94)	0.71 (0.52–0.96)	0.74 (0.54–0.97)	0.87 (0.77–0.99)	0.88 (0.79–0.98)	0.91 (0.81–0.99)
Exposure at 4–12 months	0.77 (0.67–0.89)	0.79 (0.69–0.93)	0.80 (0.72–0.92)	0.96 (0.91–1.01)	0.97 (0.92–1.03)	0.95 (0.89–1.01)
**Ampicillin**						
No exposure	Ref.	Ref.	Ref.	Ref.	Ref.	Ref.
Exposure at 0–12 months	1.08 (0.93–1.26)	1.07 (0.91–1.36)	1.08 (0.93–1.26)	1.11 (1.05–1.18)	1.15 (1.07–1.17)	1.12 (1.03–1.19)
Exposure at 0–3 months	0.93 (0.67–1.28)	0.95 (0.68–1.29)	0.96 (0.74–1.35)	1.19 (1.04–1.35)	1.14 (1.03–1.38)	1.17 (1.05–1.39)
Exposure at 4–12 months	1.21 (1.02–1.43)	1.14 (1.01–1.25)	1.27 (1.08–1.46)	1.11 (1.03–1.19)	1.16 (1.08–1.21)	1.13 (1.05–1.18)
**Ampicillin/sulbactam**						
No exposure	Ref.	Ref.	Ref.	Ref.	Ref.	Ref.
Exposure at 0–12 months	0.87 (0.60–1.24)	0.85 (0.55–1.22)	0.88 (0.61–1.27)	1.25 (1.11–1.41)	1.26 (1.12–1.44)	1.23 (1.09–1.36)
Exposure at 0–3 months	0.39 (0.10–1.58)	0.38 (0.09–1.55)	0.41 (0.12–1.62)	1.36 (0.99–1.87)	1.38 (0.98–1.86)	1.37 (0.97–1.88)
Exposure at 4–12 months	0.94 (0.65–1.37)	0.96 (0.63–1.35)	0.95 (0.64–1.36)	1.24 (1.08–1.41)	1.25 (1.07–1.43)	1.23 (1.05–1.44)

Abbreviations: Ref., reference group; aHR, adjusted hazard ratio; 95% CI, 95% confidence interval. ^a^ Adjusted hazard ratios and 95% confidence intervals derived from multivariable Cox regression models adjusted for maternal and childhood covariates and area-based socioeconomic status. ^b^ Analyses of cohort of children with prior respiratory tract infections and no other infection type. ^c^ Analyses of cohort of children without admission for more than 7 days.
